# Mistakes of Physicians

**Published:** 1891-05

**Authors:** 


### EDITORIAL.

### Mistakes of Physicians.

It is a happy thing to know that human beings are not perfect in everything that is done, and it is especially gratifying to know this because it gives the dental practitioner a great degree of relief from the exacerbations that are sometimes cast upon him by the medical practitioner. That mistakes occur by both is not to be disputed. We believe the person who knows most, generally, and can put his knowledge into practice is the best educated man, though in a specialty a specialist will far outstrip him. But there are common mistakes that physicians make that the dentist can not help but laugh at. We do not say that all physicians make these mistakes.

It is not uncommon to find physicians who do not know how many teeth an adult should have, and it is still more frequent that the number of deciduous is not known.

A very common mistake, likely to be made by a physician, is when a child with an impediment of speech is brought to him and the doctor declares it is "all due to a high roof of the mouth," when the cause is really cleft palate or a short frænum linguæ or "tongue-tie." We have actually seen physicians who did not recognize either cause. A case in practice will illustrate. A lady brought a four years old child to our office, to have an aching tooth extracted. The ache was relieved by proper remedy. On examining the mouth, however, a serious tongue-tie was noticed, and this rendered the words of the child unintelligible. The tongue could not be lifted at its tip above the incisive edge of the incisor teeth. On account of the incoherent talk and inability to walk the child was declared to be an idiot, by a physician of some thirty years practice. Imagine the suspense and anxiety of the mother over that child's future. Another physician examined the spine and legs of the child, and though weak, were developing slowly. Time and exercise would soon see the child walking, but it remained for the dentist to discover the condition of the tongue and inform the mother that the child was not an idiot. Subsequent facts support the latter's declaration.

Another source of mistakes may be found in simple neuralgic pains from an exposed pulp of a tooth. Every dentist can relate circumstances of Dr. So and So who treated Mrs. Blank for neuralgia for several months and the whole trouble was relieved in a few minutes by proper care of the teeth.

We recently saw a surgeon tie a broken jaw together with one strand of silk thread. The fracture was between the bicuspid and cuspid tooth, lower jaw. The thread was passed around the labial sides of the incisors to the second bicuspids and then across the mouth. The mouth was then closed and a paste board splint made which was tightly held in place by bandages. The patient was then told to "suck his food through a straw." We pitied the patient but were powerless to act. Such surgery is deplorable. An angle splint would have made life worth living in this case.

How frequently physicians mistake a common gum boil for a tumor and label it malignant. We have heard of such errors being made by "great" medical authority. Catarrh has drawn many victims to the grave by the dread of its name, and doctors are responsible, when the whole affair might have been cured by treatment of the antrum of Highmore. These and other instances indicate the small amount of intelligence displayed in regard to the mouth by physicians and surgeons. We might add that some very good physicians and surgeons have made blunders in the directions we have indicated. As for the remedy of such a state of affairs, education alone is the desideratum.

It may be said to the praise of a number of the medical colleges, especially of the west, that a department of dental surgery is embraced in the curriculum of study, and we have no fears of the graduates from such institutions making egregious mistakes of diagnosis when such institutions have for teachers some of the best men of our profession. W.

